# Conservative treatment using laser diode and systemic chemotherapy for early‐stage bilateral retinoblastoma: A 14‐year prospective cohort study

**DOI:** 10.1002/cnr2.1919

**Published:** 2023-10-17

**Authors:** Thi Anh Thu Phan, Alexis Derumigny, Minh Cuong Duong, Laurence Desjardins, Tuyet Anh Cung, Cong Kiet Nguyen

**Affiliations:** ^1^ Department of Ophthalmology University of Medicine and Pharmacy at Ho Chi Minh City Ho Chi Minh City Vietnam; ^2^ Department of Applied Mathematics Delft University of Technology Delft the Netherlands; ^3^ Faculty of Medicine, School of Population Health UNSW Kensington New South Wales Australia; ^4^ Department of Ophthalmology Institute Curie Paris France; ^5^ Department of Oncology University of Medicine and Pharmacy at Ho Chi Minh City Ho Chi Minh City Vietnam

**Keywords:** bilateral retinoblastoma, conservative treatment, retinoblastoma, systemic chemotherapy, transpupillary thermotherapy

## Abstract

**Background:**

Solid evidence of the safety and effectiveness of retinoblastoma (RB) conservative treatment using thermotherapy and systemic chemotherapy with long‐term follow‐up is scarce, especially in low‐resource countries.

**Aims:**

This study examined the outcomes of this treatment and associated predictors in Vietnam to strengthen the current RB treatment protocol focusing on preserving eye and vision in low‐resource settings.

**Methods and results:**

A prospective cohort study was conducted at Ho Chi Minh City Eye Hospital in Vietnam from 2005 to 2019. All eligible patients with bilateral RB (one eye already removed and another eye classified as group A or B) and without previous treatment were recruited. All patients received thermotherapy and six cycles of systemic three‐agent chemotherapy repeated every 4 weeks. A standardized questionnaire was used to collect information on study participants' age, symptoms, tumor characteristics, treatment, and outcomes. Among 50 eyes of all 50 patients with a median age of 9 (4–20) months, 34 eyes were in group B (68%). The median follow‐up time was 60 (60–84) months. All 139 preserved tumors regressed mostly to type 4 (70.4%) and type 3 (23.7%) scars. Kaplan–Meier analysis found the overall globe‐salvage rate at 5 years of 91.9% (95% CI: 80.1%–97.7%). Most eyes (41/50, 82%, 95% CI: 69.2%–90.2%) had a final visual acuity ≥0.1. The visual acuity is higher when tumors regressed to a type 4 scar (*p* = .007, AOR = 8.098, 95% CI: 1.79–36.53) which also shows less enucleation than a type 3 scar (*p* = .002, AOR = 0.06, 95% CI: 0.01–0.37%). Gender effect on visual acuity after treatment was significant and may be due to discrimination. No major complications were recorded.

**Conclusion:**

Conservative treatment of early‐stage RB is safe and effective. Long‐term, thorough follow‐ups of patients post‐treatment are needed. The regression patterns of scars could be a useful indicator of treatment failure.

## 1 INTRODUCTION

Retinoblastoma (RB), the most common eye cancer of childhood, is fatal if left untreated.^1^ Retinoblastoma accounts for 3% of all childhood cancers^2^ with a constant incidence of 1:15000–1:20000 live births worldwide.^3^ Retinoblastoma occurs most often in under 5‐year‐old children, especially under the age of two.^2^ It has been documented that the survival rate is around 30% in low‐income countries, 60% in lower‐middle‐income countries, 75% in upper‐middle‐income countries, and 95% in high‐income countries.^4^ Recently, this rate has been reported as high as 96.1% in an upper‐middle‐income country.^5^ Retinoblastoma treatment outcomes have improved globally over the past four decades, but large disparities between higher‐income and lower‐income countries still persist with some areas having major survival gaps.^6^ A recent international, multicenter, and registry‐based analysis of RB management has revealed that lower national income levels are associated with significantly higher rates of metastasis‐related mortality, local treatment failure, and lower globe salvage.^7^ Detecting RB at early stages to provide timely treatment is crucial in preventing blindness, cosmetic deformities, and death.^8^ The International Classification of Retinoblastoma divides intraocular RB into five groups (A‐E) to enable a better prediction of treatment success and to help choose the best treatment method.^2^, ^9^ Focal therapy selection depends on the RB group, which encompasses several characteristics including the severity of cancer, the tumor size, and position.^10^ With early‐stage RB of groups A and B, if tumors are still small and not in the peripheral retina, the most common focal therapy is transpupillary thermotherapy using laser diode with or without systemic chemotherapy.^2^ It has been documented that other local therapies have been being developed for ocular salvage in RB treatments.^2^ However, these treatments are not always available and feasible in developing countries, including Vietnam because of the lack of healthcare resources and their high cost.^11^ Given these reasons, the focal laser diode combined with systemic chemotherapy remains the main method in RB conservative treatment, especially in Vietnam. Although there are studies on the safety and effectiveness of local laser treatment combined with systemic chemotherapy worldwide, most studies either were conducted in non‐Asian populations, had short follow‐up duration, or used one or two anti‐cancer agents.^12^, ^13^, ^14^ The treatment has shifted toward vision salvage and rehabilitation in resource‐rich countries, whereas resource‐poor countries still struggle with the provision of access to primary treatment to save lives.^11^ Given these reasons, to strengthen the current RB treatment protocol that focuses on preserving eye and vision in Vietnam and comparable countries, this study was conducted to evaluate the treatment outcome of bilateral RB patients undertaking local laser treatment combined with systemic three‐agent chemotherapy and associated predictors.

## 2 METHODS

### 2.1 Setting of the study

A 14‐year prospective cohort study was conducted on a dynamic population at Ho Chi Minh City Eye Hospital between January 2005 and January 2019. Eligible patients included children with bilateral RB who had one eye already removed and the remaining eye at an early RB stage (group A and B) that had an indication of conservative treatment using laser diode 810 nm (IRIDEX Oculight® SL) combined with systemic chemotherapy. Patients who had already received treatment before the start of the study were excluded.

Histopathology of the removed eyes was performed to ensure RB diagnosis and that there was no extension of the disease outside the eye globe, that is, no scleral invasion nor any tumor cells observed at the cutting edge of the optic nerve. Therefore, those with advanced disease were excluded. According to the local treatment protocol, systemic chemotherapy is indicated in case of RB with invasion of the optic nerve, choroid or sclera, and bilateral RB. Moreover, systemic chemotherapy helps reduce tumor volume to facilitate the use of local treatments and avoid the long‐term effects of radiation therapy.^2^ Therefore, all patients in our study received six cycles of systemic three‐agent chemotherapy including vincristine (1.5 mg/m^2^ intravenous for 15 min, 0.05 mg/kg for patients <36 months of age, maximum dose 2 mg, day 0), carboplatin (560 mg/m^2^ in 120 cc/m^2^ dextrose 5% in 1/4 normal saline intravenous for 60 min, 18.6 mg/kg for patients <36 months of age, day 0), and etoposide (150 mg/m^2^ in 150 cc/m^2^ dextrose 5% in 1/4 normal saline intravenous for 60 min, 5 mg/kg for patients <36 months of age, days 0 and 1 of each course) repeated at an interval of 4 weeks at Ho‐Chi‐Minh City Cancer Hospital.^15^

Local laser diode was used for the preserved eye at the same time of enucleation of the worse eye if the tumors did not reach the macula and optic nerve, or after two chemotherapy cycles following enucleation if the tumors covered these areas. After completing six cycles of chemotherapy, new tumors or recurrences occurring during follow‐up were treated with only laser diode. Cryotherapy, which is another local treatment, was used for those tumors when their locations were too peripheral to be reached by laser diode. The treatment with laser diode or cryotherapy was repeated every 4 weeks until either the tumors regressed, or the eye was removed in case when the tumors progressed toward the anterior chamber or when vitreous seeds appeared.

Given that RB is a rare pediatric cancer, all eligible patients were invited to participate in the study during the study period. Enrolment of each participant began either at the start point of the study if they started treatment at this time or on the enrolment date of that patient if they were referred to our hospital after the study had begun. All participants were followed up monthly in the first year, every 2 months in the second year, every 3 months in the third year, every 4 months in the fourth year, and every 6 months thereafter until the end of the study (to ensure that each participant was followed up during at least 5 years) or until loss to follow‐up either due to death, referral, or removal of the preserved eye. All patients' direct caregivers provided written informed consent to participate in the study. The study was approved by the Ethics Committee of the University of Medicine and Pharmacy at Ho Chi Minh City (approval number 360/ĐHYD‐HĐĐĐ).

A standardized questionnaire was used to collect information on participants' age, gender, clinical symptoms, tumor characteristics, laser diode parameters, regression patterns of scars, and outcomes. Tumor characteristics included the number of tumors, the longest base diameter (in comparison with the optic disc diameter on the fundus image), and position (in the central retina, which is less than 1.5 mm from the optic disc or 3.0 mm from the fovea; or outside the central retina). Laser diode parameters included laser power and the total duration of laser treatment across all treatment sessions. Regression patterns of scars included type 0 (no visible remnant), type 1 (totally calcified remnant), type 2 (non‐calcified remnant), type 3 (partially calcified remnant), and type 4 (flat scar).^16^, ^17^, ^18^ Treatment outcomes included the number of eyes that were preserved, recurrences, complications, and visual acuity post‐treatment. Children who were at least 3 years of age and cooperative were assessed for corrected visual acuity at each follow‐up using the Snellen or Tumbling E vision chart. For those who were under 3 years old or uncooperative, the visual acuity was estimated by using the “central, steady, and maintained” qualitative method.^19^, ^20^ In detail, “central” refers to the corneal reflex from a fixation light falling at the center of the pupil. Steadiness of the fixation is assessed while moving the light source, and nystagmus denotes unsteady fixation. Maintenance of fixation refers to the ability to keep the eye fixed on a target. A rough estimation of the visual acuity in case of central fixation is at least 0.1, while visual acuity is estimated to be less than 0.1, in case of unsteady central fixation. All clinical examinations, measurements, and treatments were performed by qualified ophthalmologists and oncologists at our study clinic.

### 2.2 Statistical analysis

Data were managed and analyzed using the statistical software R.^21^ Continuous variables were displayed with their respective median, upper (UQ), and lower (LQ) quartile. Categorical variables are presented as count data with their share in the sample. The Kaplan–Meier method was used to estimate the rate of eye conservation and its 95% confidence region was computed using the R package “BPCP”.^22^ Multivariate logistic regression models were developed to test for predictors of eye removal and visual acuity. Coefficients $\beta_{i}$ of the predictors were estimated using the “glm” function in R and odds ratios were computed by $\text{OR}=\exp\left(\beta \right)$. 95% confidence intervals (95% CIs) were computed for the point estimates of odds ratios for comparison. These 95%CIs on the OR were computed by $95\%\mathrm{CI}=\left[\exp\left(\hat{\beta }-1.96\times \text{stderr}\right),\exp\left(\hat{\beta }+1.96\times \text{stderr}\right)\right]$, where stderr is the standard error on the estimated coefficient $\hat{\beta }$. Note that these intervals are quite large due to the low sample size of the study and the exponential function. Testing was performed at the usual level α=5%.

## 3 RESULTS

### 3.1 Characteristics of study participants, tumors, and treatments

The recruitment rate was 100% and thus, a total of 50 eyes of 50 patients with 139 tumors were included in this study. The median age is 9 (4–20) months. The median follow‐up time was 60 (60–84) months. Most preserved eyes (68%) were in group B (Appendices S1 and S2). Only eight eyes (16%) developed leukocoria, all of which belonged to group B. Among 139 tumors, 95 (68%) were detected on admission and 44 (32%) were newly developed afterwards. Most tumors (76%) were located outside the central retina and had a median longest diameter of 3.0 (2.0–4.0) mm. The median laser power used was 600 (400–800) mW, and the median total duration of treatments was 5.0 (2.0–20.5) minutes (Table 1).

**TABLE 1:** Demographic, clinical, and treatment characteristics of 50 patients receiving conservation therapy.

Characteristics	Statistics^a^
Age (months)	9 (4–20)
Follow‐up period (months)	60 (60–84)
Gender (*N* = 50)	
Male	30 (60)
Female	20 (40)
Preserved eyes (*N* = 50)	
Group A	16 (32)
Group B	34 (68)
Clinical signs (*N* = 50)	
Leukocoria	8 (16)
No sign	42 (84)
Tumor (*N* = 139)	
Primary tumors detected on admission	95 (68)
New tumors developed after admission	44 (32)
Position of tumor (*N* = 139)	
Center of the retina (≤3.0 mm from the macula or ≤1.5 mm from the optic nerve)	33 (24)
Outside the central retina (>3.0 mm from the macula and >1.5 mm from the optic nerve)	106 (76)
Diameter of the tumor (mm)	3.0 (2.0–4.0)
Laser power (mW)	600 (400–800)
Total laser treatment duration (minutes)	5.0 (2.0–20.5)

^a^ Categorical variables are presented as *n* (%), while continuous variables are presented as median (LQ – UQ).

A relapse was documented in 12 tumors (8.6%) of which eight were treated only with laser diode and preserved (Table 2). The remaining four recurrent tumors of four eyes were treated using laser diode combined with cryotherapy, one was preserved, and three were removed. In our study, only 135 regression scars were recorded from 139 tumors because one eye having four persistent tumors was removed after 6 months due to unresponsiveness to treatment. There were eight (6%) type 2 scars, 32 (24%) type 3 scars, and 95 (70%) type 4 scars. No type 0 and type 1 scars were recorded during follow‐up, nor were any major complications. Six preserved eyes (12%) developed vitreous fibrosis, and four (8%) developed vitreous hemorrhage and vitreous seeding. Other complications such as local cataract, local iris atrophy, corneal opacity, iris adhesions, retinal detachment, or vascular occlusion were not detected. Distant metastases and prolonged sequelae of chemotherapy such as deafness, leukemia, and renal failure were not recorded during the follow‐up. Four eyes (8%) were removed including three (6%) group B eyes and one (2%) group A eye. Five patients with follow‐up time from 2 to 4 years, dropped out of the study having stable scars, no new tumor, recurrence, or complication until their last follow‐up. Most eyes (41/50, 82%) had a final vision of at least 0.1 (in decimal).

**TABLE 2:** Clinical outcomes of 50 patients receiving conservative treatment.

Characteristics	Statistics^a^
Tumors after treatment (*N* = 139)	
Regressed	123 (88.5)
Persistent	4 (2.9)
Recurrent	12 (8.6)
Treatment of tumor recurrence (*N* = 12)	
Laser diode	8 (67.0)
Laser diode with cryotherapy	4 (33.0)
Regressed scars (*N* = 135)	
Type 0	0 (0.0)
Type 1	0 (0.0)
Type 2	8 (6.0)
Type 3	32 (24.0)
Type 4	95 (70.0)
Enucleation (*N* = 50)	
Group A	1 (2.0)
Group B	3 (6.0)
Complications (*N* = 50)	
Vitreous fibrosis	6 (12.0)
Vitreous hemorrhage and vitreous seeding	4 (8.0)
No complication	40 (80.0)
Final visual acuity^b^ (*N* = 50)	
At least 0.1	41 (82.0)^c^
Less than 0.1	9 (18.0)

^a^ Categorical variables are presented as *n* (%).

^b^ Visual acuity of the eyes that were removed was 0. For those who were lost to follow‐up, visual acuity was recorded at the last follow‐up. Visual acuity of the remaining eyes was measured after at least 5 years of follow‐up.

^c^ Including one eye preserved with cryotherapy in combination with laser diode and chemotherapy as the related tumors spread to the most peripheral retina.

### 3.2 Eye conservation rate

**FIGURE 1:**
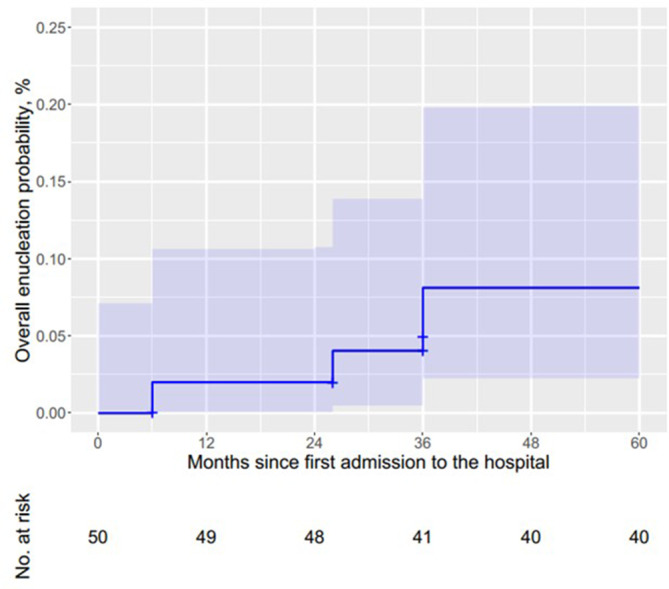
Overall enucleation rate* (blue line, estimated by Kaplan–Meier) and its 95% confidence region (shaded area). *The calculation of this rate included 50 eyes. Of these eyes, one eye in which the related tumors spread to the most peripheral retina, and thus was preserved with laser diode and systemic chemotherapy combined with cryotherapy.

A Kaplan–Meier analysis estimated that the overall globe‐salvage rate after the first and second year was 98% (Figure 1), which decreased to 96% and 92% after 26 and 36 months, respectively.

### 3.3 Predictors of eye removal

Multivariate logistic regression showed that when the tumor regressed to a type 3 scar, the possibility of eye removal was 17 times higher than that of a type 4 scar (*p* = .002, AOR = 0.06, 95% CI: 0.01–0.37) (Table 3). Tumor diameter (*p* = .15), age (*p* = .09), gender (*p* = .94), and position of the tumor (*p* = .82) were not significantly associated with the probability of eye removal.

**TABLE 3:** Model for the prediction of enucleation.

Variable	*p*‐value	Adjusted odds ratio	95% CI
Constant	.18	6.51	0.42–100.9
Tumor diameter	.15	0.67	0.38–1.16
Age	.09	0.86	0.72–1.02
Gender (female)	.94	0.95	0.22–3.96
Tumor position			
Center of the retina	.82	0.83	0.15–4.23
Outside the central retina^a^	–	1	–
Regressed scar			
Type 2	.99	0.00	0.00–∞
Type 3^a^	–	1	–
Type 4	**.002**	0.06	0.01–0.37

*Note*: The bold number refers to the *p* value that is significant (smaller than the usual level 0.05).

^a^ Reference group.

### 3.4 Predictors of visual acuity post‐treatment

Multivariate logistic regression showed that the probability of visual acuity of at least 0.1 was six times higher in male gender (*p* = .002, AOR = 0.18, 95% Cl: 0.06–0.56) and 8 times higher when the tumor regressed to a type 4 scar compared with those regressed to a type 3 scar (*p* = .007, AOR = 8.098, 95% CI: 1.79–36.53) (Table 4). Tumor diameter (*p* = .15), age (*p* = .09), and position of the tumor (*p* = .82) were not significantly associated with visual acuity.

**TABLE 4:** Model for the prediction of visual acuity.

Predictive factors	*p*	Adjusted OR	95% CI
Constant	.91	0.89	0.10–7.55
Tumor diameter	.06	1.42	0.98–2.05
Age	.66	1.01	0.95–1.07
Gender (female)	**.002**	0.18	0.06–0.56
Tumor position			
Center of the retina	.10	0.41	0.13–1.22
Outside the central retina^a^	–	1	–
Regressed scar			
Type 2	.99	5.13 × 10^7^	0.000–∞
Type 3^a^	–	1	–
Type 4	**.007**	8.098	1.79–36.53

*Note*: The bold number refers to the *p* value that is significant (smaller than the usual level 0.05).

^a^ Reference group.

## 4 DISCUSSION

The age distribution of our study participants is comparable to other similar studies worldwide, confirming the high burden of RB among children less than 2 years old.^23^, ^24^ Our median follow‐up time is also comparable to other available long‐term studies.^13^, ^18^, ^25^ We found that the proportion of RB group A was lower than that of group B. This finding concurs with an Indian study in which there were more group B eyes (88.2%, 95% CI: 81.2–92.9) than group A eyes (11.8%, 95% CI: 7.1–18.8).^26^ The low proportion of group A tumors may be due to the late detection of RB or the small tumor size and their remoteness from the central retina in this group, which makes them difficult to detect. All patients in our study had already one eye removed due to the very advanced stage of RB. As a result, the second eye might develop large tumors. Group B tumors are usually located in the central retina or sufficiently big to be detectable by not only clinical examination, but also clinical signs like leukocoria and strabismus.^27^ We found eight preserved eyes that exhibited leukocoria only, and all these eyes belonged to group B. Since leukocoria was the only sign that was detected in the preserved eyes, we believe that it should be used for RB screening purposes. Therefore, in order to facilitate timely conservative treatment, a more rigorous screening program is needed to detect RB at early stages based on leukocoria. However, it should be noted that leukocoria may also be a symptom of pseudoretinoblastoma. Indeed, Shields et al.^28^ recorded 22%, and Mirzayev et al.^29^ recorded 28.4% of cases with leukocoria that actually had pseudoretinoblastoma including Coats' disease, PFV, and vitreous hemorrhage. Considering this, patients should immediately be referred to a pediatric ophthalmologist after the detection of leukocoria for timely diagnosis and treatment.

We found that 32% of 139 tumors receiving conservation therapy newly developed during follow‐up. This finding is consistent with a study conducted in the USA (34%, 95% CI: 25.7–43.4) with a median follow‐up of 5 years.^24^ Another study found that a new growth of intraocular RB can occur more than 10 years after the eye responds to conservative treatment.^30^ Therefore, a long‐term follow‐up of RB eyes, especially bilateral RB that receives conservation therapy, is essential. In addition, similar to another report,^26^ we found that most tumors were located outside the central retina. In light of this, the area outside the central retina should also receive more attention during the follow‐up.

The median longest diameter of all tumors in our study is comparable to that of other studies.^12^, ^13^, ^26^ However, our median laser power was higher, while the median treatment duration was shorter than those in other studies worldwide.^13^, ^23^, ^31^ This may be because we only included early‐stage tumors, while some authors selected tumors at all stages. In addition, we used slightly higher energies and thus reduced the treatment duration. Gündüz et al. found that after intravenous chemotherapy, recurrence developed in 40 eyes (16.3%), new tumors in 29 eyes (11.8%), and both recurrence and new tumors in 24 eyes (9.8%).^32^ Dalvin et al. followed 556 eyes and revealed main solid tumor recurrence (*n* = 355, 64%), and new tumors with or without recurrence (*n* = 118, 21%).^33^ Our rate of tumor recurrence (9%) was lower than that of other studies which included more advanced RB and used only primary intravenous chemotherapy.^23^, ^32^, ^33^, ^34^ The most common treatment methods used for recurrent or new tumors include cryotherapy, transpupillary thermotherapy, and intra‐arterial chemotherapy.^32^ The predominant patterns of regressed scars in our study were types 3 and 4, which were similar to those of other studies.^17^, ^23^, ^26^, ^34^ No serious complications related to laser treatment such as retinal detachment, vascular occlusion, local cataract, local iris atrophy, corneal opacity, and iris adhesions were detected in a study in India.^26^ However, in another study with more advanced RB patients, these serious complications were documented with a low frequency.^23^ We noticed six eyes (12%) developed vitreous fibrosis, four eyes (8%) developed vitreous hemorrhage and vitreous seeding, of which these four eyes were removed because of treatment unresponsiveness. A Kaplan–Meier analysis estimated that the overall globe‐salvage rate after 5 years in our study is as high as those reported by Schueler et al.^23^ (94%, 33/35, 95% CI: 81.4–98.4) and Chawla et al.^26^ (93.3%, 111/119, 95% CI: 87.3–96.6), in which thermotherapy combined with systemic chemotherapy was also used. A total of four eyes were removed during our study including three group B eyes and one group A eye. Our finding is similar to an Indian study in which eight group B eyes (8/119, 6.7%) developed treatment failure, while all group A eyes (14/119, 11.8%) responded to treatment.^26^ These findings confirm that the combination therapy of local treatment and systemic chemotherapy could be a safe and effective treatment of early‐stage RB in low‐resource settings.

Regarding predictors of treatment failure, Chawla et al. found an association between treatment failure and larger tumors as well as proximity to the posterior pole.^26^ We did not record any direct association between eye removal and tumor diameter, age, gender, and tumor position. Such a lack of significant effect could result from the small sample size considered in this study. However, we found that regression patterns of scars were a predictor of eye removal after receiving conservative therapy; to the best of our knowledge, this finding has not yet been reported elsewhere.

Most eyes in our study (82%) had a final vision of at least 0.1. Demirci et al. conducted a study in which the conservation therapy and follow‐up time were comparable to ours and found that 67% (36/54, 95% CI: 53.4–77.8) of eyes had a final vision of at least 0.1.^25^ Another study with a comparable mean follow‐up period found that 76% (28/37, 95% CI: 60.0–86.6) of study participants had a visual acuity above 0.1, which is similar to our findings.^35^ Regarding predictors of visual acuity post‐treatment, a study examining the effectiveness of different RB conservative therapies found that tumor location is an important predictor of good visual acuity after treatment.^36^ Another study also found that tumor diameter and its position close to the macula are the most significant predictors of vision loss following treatment.^37^ However, we did not record any significant effect of tumor size and position on visual acuity. This could be explained by the fact that the macula is the center of vision.^38^ Therefore, even a small tumor located in the macula can cause serious vision loss. In contrast, a tumor is located in the peripheral retina with poor response to the treatment can extend to the central retina and affect the vision.

Nevertheless, we found that gender and regression patterns of scar were significant predictors of visual acuity after receiving conservative therapy that has not yet been reported elsewhere. In light of our findings, the regression patterns of scars could be used as a predictor of treatment failure and visual acuity post‐treatment. However, the precise value of the effect of these predictors remains an open question, as our confidence intervals are quite wide. Recently, a study about the survival disadvantage of male children with RB in the United States showed a sex differential in survival with a higher risk of dying identified among males compared with females, which may be explained in part by male X‐linkage.^39^ But no evidence of sex predilection in RB was found in a study which is estimated to include over half of new RB patients worldwide.^40^

A high male‐to‐female ratio in Asian countries, India in particular, which may have had an impact on global‐level analysis, is likely due to gender discrimination in access to healthcare in these countries, rather than a biological difference between sexes.^40^ This could be also the explanation for our findings that there are more male patients in our study (male: female ratio is 1.5) and that male gender had an effect on visual acuity after treatment. In Vietnam, especially in the rural areas, the old style of mentality about preferring to have a male child still exists. Indeed, Vietnam has an extreme imbalance in the sex ratio at birth due to son preference and sex‐selective abortion.^41^ The Census (2019) reported that the sex ratio at birth was as high as 111.5 boys born for every 100 girls.^42^ For the same reasons, male children might receive more attention in their families, which can lead to earlier treatment and better compliance during the conservative treatment period and long‐term follow‐up.

Our study has some limitations. Firstly, as this study was conducted at only one center, the generalizability of the findings may be limited. However, our hospital is the leading tertiary teaching hospital for pediatric ophthalmology in southern Vietnam and receives patients from not only Ho Chi Minh City, but also from half of the country including the the Mekong delta. Secondly, we were unable to prevent missing data due to loss of follow‐up because of patients' difficulties in traveling between our hospital and the rural areas where they lived. Although we did not record any deaths, five cases had missing data. However, all tumors of these five cases had regressed very well at the last follow‐up. Nevertheless, our study is among the few recent studies in this field with a considerably long follow‐up period in Vietnam and worldwide. Our study secured a minimum follow‐up of 5 years for all participants. The small sample size is also a limitation of our study. Therefore, we recommend the organization of a future study at a higher scale, potentially involving different centers in several countries in order to get better estimates of the best treatment. The difficulty of such a follow‐up would be related to the differences in the health systems, especially in the local treatment guidelines. Even if valuable, such a large‐scale study may be difficult to conduct due to financial, logistic, and legal reasons. Hence, another solution is to perform other studies in the context of Vietnam, for example in the framework of a national project gathering the energy of all specialized hospitals in the country. Such a larger sample size would definitely help increase the accuracy of our estimations.

In conclusion, the focal laser diode combined with systemic chemotherapy may be used as a primary therapy for RB tumors at early stages in Vietnam and in other comparable low‐resource settings. Given the occurrence of newly developed tumors even after successful treatment, a long‐term thorough follow‐up of patients post‐treatment is needed. Follow‐up examinations should not overlook the peripheral retina. The regression patterns of scars could be used as a predictor of treatment failure and visual acuity post‐treatment.

## AUTHOR CONTRIBUTIONS

**Thi Anh Thu Phan:** Conceptualization (equal); data curation (equal); formal analysis (equal); investigation (equal); methodology (equal); project administration (equal); resources (equal); software (equal); validation (equal); visualization (equal); writing – original draft (equal); writing – review and editing (equal). **Alexis Derumigny:** Conceptualization (equal); data curation (equal); formal analysis (equal); resources (equal); software (equal); validation (equal); visualization (equal); writing – original draft (equal); writing – review and editing (equal). **Minh Cuong Duong:** Conceptualization (equal); data curation (equal); formal analysis (equal); methodology (equal); supervision (lead); validation (equal); writing – original draft (equal); writing – review and editing (equal). **Laurence Desjardins:** Conceptualization (equal); data curation (equal); investigation (equal); methodology (equal); validation (equal); writing – review and editing (equal). **Tuyet Anh Cung:** Formal analysis (equal); investigation (equal); methodology (equal); resources (equal); writing – review and editing (equal). **Cong Kiet Nguyen:** Conceptualization (equal); data curation (equal); formal analysis (equal); investigation (equal); methodology (equal); project administration (equal); resources (equal); validation (equal); writing – review and editing (equal).

## CONFLICT OF INTEREST STATEMENT

The authors have stated explicitly that there are no conflicts of interest in connection with this article.

## Supporting information


**Appendix S1** RB eye Group A: before (1) and after (2) conservative treatment.

**Appendix S2** RB eye Group B: before (1) and after (2) conservative treatment.

## Data Availability

The datasets used and/or analyzed during the current study are available from the corresponding author on reasonable request
