# Supplementary figures and images for: Conservative treatment using laser diode and systemic chemotherapy for early‐stage bilateral retinoblastoma: A 14‐year prospective cohort study
Source: Cancer Rep (Hoboken). 2023 Oct 17;7(1):e1919. doi: 10.1002/cnr2.1919 (PMC10809195; doi:10.1002/cnr2.1919)

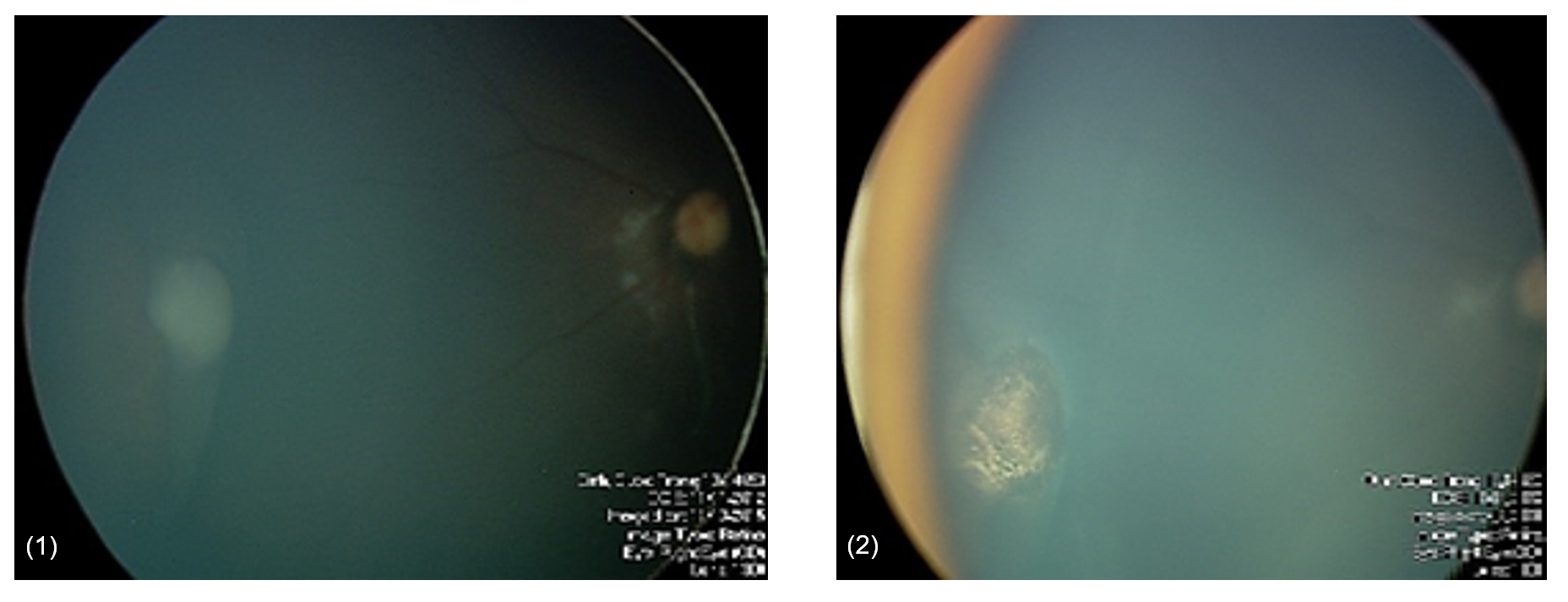

Supplement: Supplementary file 1 — Appendix S1 RB eye Group A: before (1) and after (2) conservative treatment. [file CNR2-7-e1919-s001.jpg]

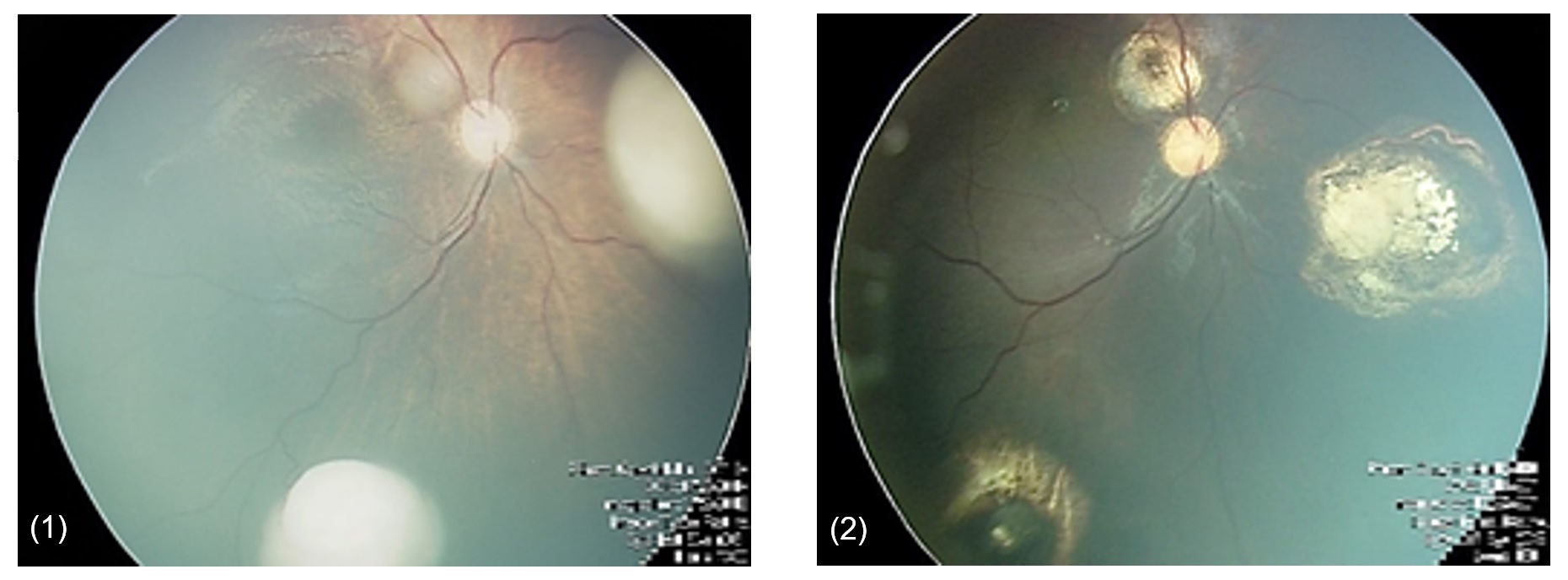

Supplement: Supplementary file 2 — Appendix S2 RB eye Group B: before (1) and after (2) conservative treatment. [file CNR2-7-e1919-s002.jpg]
